# Tetherin Inhibits Cell-Free Virus Dissemination and Retards Murine Leukemia Virus Pathogenesis

**DOI:** 10.1128/JVI.02286-16

**Published:** 2017-05-26

**Authors:** Rachel A. Liberatore, Emily J. Mastrocola, Chelsea Powell, Paul D. Bieniasz

**Affiliations:** a Aaron Diamond AIDS Research Center, New York, New York, USA; b Howard Hughes Medical Institute, Chevy Chase, Maryland, USA; c The Rockefeller University, New York, New York, USA; University of Utah

**Keywords:** tetherin, retroviruses, vesicular stomatitis virus, viral pathogenesis

## Abstract

The relative contributions of cell-free virion circulation and direct cell-to-cell transmission to retroviral dissemination and pathogenesis are unknown. Tetherin/Bst2 is an antiviral protein that blocks enveloped virion release into the extracellular milieu but may not inhibit cell-to-cell virus transmission. We developed live-cell imaging assays which show that tetherin does not affect Moloney murine leukemia virus (MoMLV) spread, and only minimally affects vesicular stomatitis virus (VSV) spread, to adjacent cells in a monolayer. Conversely, cell-free MLV and VSV virion yields and VSV spread to distal cells were dramatically reduced by tetherin. To elucidate the roles of tetherin and cell-free virions during *in vivo* viral dissemination and pathogenesis, we developed mice carrying an inducible human tetherin (hTetherin) transgene. While ubiquitous hTetherin expression was detrimental to the growth and survival of mice, restriction of hTetherin expression to hematopoietic cells gave apparently healthy mice. The expression of hTetherin in hematopoietic cells had little or no effect on the number of MoMLV-infected splenocytes and thymocytes. However, hTetherin expression significantly reduced cell-free plasma viremia and also delayed MoMLV-induced disease. Overall, these results suggest that MoMLV spread within hematopoietic tissues and cell monolayers involves cell-to-cell transmission that is resistant to tetherin but that virion dissemination via plasma is inhibited by tetherin and is required for full MoMLV pathogenesis.

**IMPORTANCE** Retroviruses are thought to spread primarily via direct cell-to-cell transmission, yet many have evolved to counteract an antiviral protein called tetherin, which may selectively inhibit cell-free virus release. We generated a mouse model with an inducible tetherin transgene in order to study how tetherin affects retroviral dissemination and on which cell types its expression is required to do so. We first developed a novel *in vitro* live-cell imaging assay to demonstrate that while tetherin does indeed dramatically reduce cell-free virus spreading, it has little to no effect on direct cell-to-cell transmission of either vesicular stomatitis virus (VSV) or the retrovirus MoMLV. Using our transgenic mouse model, we found that tetherin expression on hematopoietic cells resulted in the specific reduction of MoMLV cell-free plasma viremia but not the number of infected hematopoietic cells. The delay in disease associated with this scenario suggests a role for cell-free virus in retroviral disease progression.

## INTRODUCTION

Tetherin is an antiviral gene product that inhibits the release of nascent HIV-1 particles (1, 2) and other enveloped viruses (3–7). Tetherin entraps virions at the cell surface by the partitioning of its two membrane anchors between the budding virion envelope and the plasma membrane (8–12). However, while the effects of tetherin on cell-free virus release are well established, its effects on virus transmission between cells in direct contact are less clear, as experiments conducted *in vitro* have yielded various results (13–18). Moreover, *in vitro* studies cannot recapitulate the anatomy of virus-infected tissues in an animal host.

Many studies have described the antiviral activity of tetherin *in vitro*, but far fewer have examined the effects of tetherin *in vivo*, as studies of the *in vivo* effects of tetherin are often confounded by the antiviral effects of other interferon (IFN)-stimulated genes (ISGs). In mice, tetherin is constitutively expressed on only a few cell types, but it is strongly upregulated in response to type I IFN in many cells (19). LP-BM5 murine leukemia virus (MLV) infection in mice induces an IFN response (20), and replication and pathogenesis of this virus are enhanced in tetherin-knockout mice (21). Conversely, Moloney murine leukemia virus (MoMLV) does not induce a strong type I IFN response, and tetherin knockout enhances its replication only under conditions of artificial IFN induction (21). However, a naturally occurring tetherin polymorphism that results in higher constitutive surface expression levels correlates with reduced Friend retrovirus replication in mice (22).

In the present study, we reexamined the effects of tetherin on the spread of two enveloped viruses (MoMLV and vesicular stomatitis virus [VSV]) *in vitro* by using a novel live-cell imaging analysis of the simplest model of a tissue, a cell monolayer. We also examined its effects on MoMLV dissemination *in vivo* by using mice engineered to carry an inducible human tetherin (hTetherin) transgene. We found that tetherin severely limits cell-free virus spread to distal cells in a monolayer, while the rate of dissemination to proximal cells is largely unaffected. In the case of MoMLV, the predominant mode of spread through a monolayer is via direct cell-to-cell transmission, irrespective of tetherin expression. *In vivo*, we found that ubiquitous hTetherin expression resulted in runt development and premature lethality, but mice with hematopoietic cell-restricted hTetherin expression displayed normal growth. Notably, hTetherin-expressing, MoMLV-infected mice had nearly wild-type (WT) numbers of infected cells in spleen and thymus tissues but markedly reduced plasma viremia and a delayed progression of MoMLV-induced leukemia. These results suggest that MoMLV spread within tissues and cell monolayers largely involves direct cell-to-cell transmission but that dissemination of cell-free virions via plasma is required for the full pathogenic effects of MoMLV infection.

## RESULTS

### Effects of tetherin on viral spread in cell monolayers.

To examine the effects of tetherin on cell-to-cell and cell-free retroviral dissemination *in vivo*, we first established an *in vitro* viral replication assay in which the two modes of virus spread could be measured and discriminated. For the initial development of assays in cultured cells, we took advantage of the known capacity of VSV to spread rapidly in monolayer cultures via both direct cell-to-cell contact and long-distance diffusion of cell-free virions (23). Because of this property, VSV titers are typically measured in PFU, using cell monolayers that are overlaid with soft agar soon after infection to block virion diffusion through the cell culture supernatant. Under agar, virions generated by an initially infected cell are propagated only to adjacent cells in the monolayer, generating a roughly circular plaque whose diameter increases with time.

**FIG 1:**
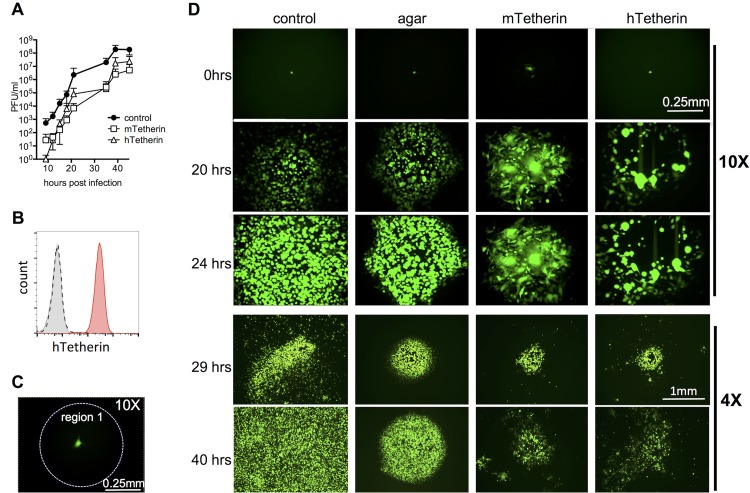
Effects of tetherin on VSV spread in a cell monolayer. (A) NIH 3T3 cells stably expressing empty vector (control) or either mouse or human tetherin (mTetherin or hTetherin) were infected with VSV (MOI = 0.0001), and viral titers (PFU per milliliter) in the culture supernatant were measured over 2 days. (B) NIH 3T3 cells were transduced with pLHCX containing hTetherin and selected with hygromycin. Single-cell clones were isolated and tested for hTetherin expression by flow cytometry. Cells were either left unstained (gray filled histogram), stained for hTetherin (red filled histogram), or stained with an isotype control antibody (dashed black line). (C) Designation of the region in which GFP fluorescence was measured to quantify viral spread for Fig. 2A and 4A. (D) Imaging of plaques after VSV-GFP infection of NIH 3T3 cells which were otherwise unmanipulated (control), were overlaid with agar after infection, or expressed mTetherin or hTetherin. The upper three rows (0, 20, and 24 h) depict a single representative focus for each condition, while the lower two rows (29 and 40 h) show different representative foci from separate experiments, at a lower magnification.

The release of cell-free VSV particles from infected cells is inhibited by tetherin (4); thus, an initial question was whether tetherin could inhibit cell-free and/or cell-to-cell virus spread in a monolayer. We generated NIH 3T3 cells stably expressing either mouse tetherin (21) or human tetherin (Fig. 1B) and confirmed that both reduced the level of virions in the cell-free culture supernatant by 10- to 100-fold during a VSV replication assay over ∼2 days (Fig. 1A). Next, we took advantage of the ability of an agar overlay to dissect the contributions of cell-free and direct cell-to-cell viral transmission to VSV spread. Specifically, we measured the dynamics of VSV spread through a cell monolayer in which virus propagation was either unrestricted or restricted to adjacent cells by an agar overlay and compared viral propagation under these two conditions to viral propagation in cells expressing tetherin. To accomplish this, we used a live-cell imaging approach. Cell monolayers were infected with green fluorescent protein (GFP)-expressing VSV at a multiplicity of infection (MOI) of 10^−4^ (∼50 PFU per 35-mm dish). After 1 h, the culture medium was replaced with either a liquid or agar overlay. The cultures were then inspected periodically for the next 6 to 8 h, and when individual GFP-positive cells were detected, they were centered in a field of observation. Infection of cells that did or did not express human or mouse tetherin proteins yielded approximately equal numbers of GFP-positive cells, consistent with previous findings that tetherin specifically inhibits virion release but not the other steps in VSV replication (4). VSV-GFP spread in the monolayer was recorded by acquiring images every 10 min for the ensuing 24 to 40 h (Fig. 1D; see Movies S1 and S2 in the supplemental material). Under each condition, VSV-GFP spread from the initially infected cell to neighboring cells formed a focus of contiguous infected cells. Additionally, under unrestricted conditions (i.e., no tetherin expression and no agar overlay), VSV-GFP infection also became evident in cells that were not in direct contact with the central focus of infection (Fig. 1D; Movies S1 and S2, upper left panels), consistent with the notion that some cells become infected via diffusion of virions in the extracellular culture medium. These distally infected cells were observed beginning at approximately 20 h postinfection and were especially evident by 29 h postinfection, and at a lower magnification (Fig. 1D). As expected, the distal population of infected cells was not observed in the presence of an agar overlay (Fig. 1D; Movies S1 and S2, upper right panels). Notably, expression of either mouse or human tetherin was also able to inhibit VSV-GFP spread to distal cells but did not appear to greatly inhibit infection of cells that were proximal to infected cells. Thus, tetherin appeared to inhibit distal propagation in a manner similar to that of the restriction imposed by an agar overlay (Fig. 1D; Movies S1 and S2, lower panels). Tetherin also appeared to enhance cell syncytium formation (which does not ordinarily occur during VSV replication at neutral pH) and cell death within infected foci. We speculate that these effects may have been caused by virion accumulation on cell surfaces in contact with neighboring cells.

**FIG 2:**
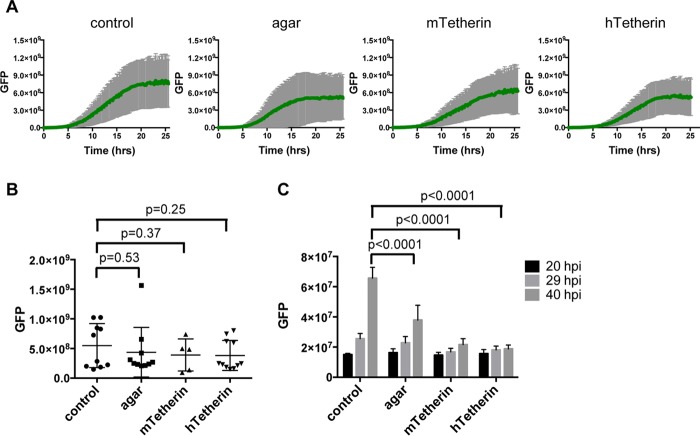
Quantitation of VSV spread in a cell monolayer. (A) Quantitation of VSV-GFP growth in region 1 under each condition. The green line represents the average integrated GFP signal for 6 to 10 movies per condition, with error bars (in gray) indicating 1 standard deviation. (B) VSV-GFP integrated intensity in region 1 at 15 h postinfection. Each dot represents one movie, with the horizontal lines showing the averages for all movies and error bars indicating 1 standard deviation. *P* values were determined by Student's *t* test. (C) Quantitation of total GFP intensity in each large, low-magnification field (∼2.5 mm × 2 mm, as depicted in Fig. 1D). Columns represent the average GFP intensities under each condition at the indicated time points, with error bars representing 1 standard deviation. *P* values were determined using Student's *t* test.

We quantified these observations by measuring the integrated GFP fluorescence in an ∼0.5-mm region corresponding to the centrally located focus or plaque (region 1) (Fig. 1C). The accumulations of GFP fluorescence within this central focus were similar under the different conditions (Fig. 2A and B), suggesting that neither an agar overlay nor tetherin expression significantly inhibited VSV transmission between cells in direct contact. In contrast, quantitation of the total GFP intensity in a larger microscopic field at various time points postinfection revealed a different pattern. By 40 h postinfection, there was significantly more GFP fluorescence in the larger field under unrestricted growth conditions than in the presence of an agar overlay or tetherin (Fig. 1D and 2C), indicating that outward VSV spread included a component that was diffusible through the culture medium to distal cells. Thus, tetherin can restrict the spread of VSV by acting on cell-free virions but does not greatly inhibit VSV spread between cells that are in direct contact in a cell monolayer.

**FIG 3:**
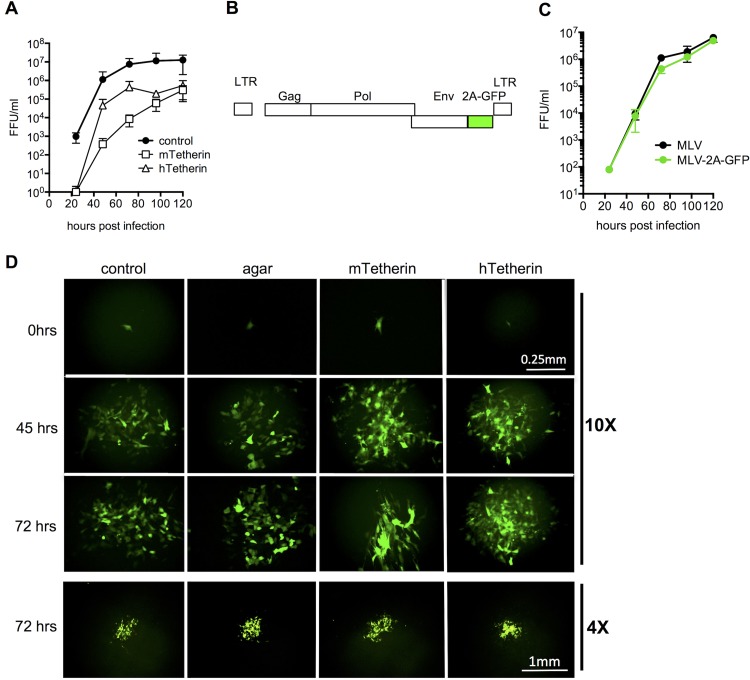
Effects of tetherin on MoMLV spread in a cell monolayer. (A) NIH 3T3 cells expressing empty vector (control) or either mouse or human tetherin (mTetherin or hTetherin) were infected with MoMLV (MOI = 0.01), and viral titers (focus-forming units [FFU] per milliliter) in the culture supernatant were measured over 5 days. (B) Schematic representation of a replication-competent MoMLV construct in which an EGFP cDNA was inserted into the pNCS molecular clone at the 3′ end of *env*, following an FMDV 2A site, generating MLV-2A-GFP. (C) Replication of MLV-2A-GFP and the parental virus generated from pNCS (MLV) in NIH 3T3 cells following infection at an MOI of 0.05. (D) NIH 3T3 cells were infected with MLV-2A-GP (MLV-GFP) as described in the legend to Fig. 1D and imaged for 72 h. The upper three rows (0, 45, and 72 h) depict a single representative focus for each condition, and the bottom row (72 h) shows different representative foci from separate experiments.

We next used the same approach to determine the mode by which MoMLV spreads through monolayer cultures and whether its spread is inhibited by tetherin. First, we confirmed that human tetherin, like mouse tetherin, is able to restrict the generation of cell-free MoMLV by infecting NIH 3T3 cells and monitoring the accumulation of infectious virions in culture supernatant over the course of 5 days. As expected, both tetherin proteins inhibited the generation of extracellular MoMLV virions (Fig. 3A).

To enable the measurement of MoMLV spread through monolayers, we constructed a replication-competent MoMLV construct that expresses GFP (MLV-GFP) in infected cells. This was accomplished by the insertion of sequences encoding a foot-and-mouth disease virus (FMDV) 2A site and GFP at the 3′ end of the *env* gene (Fig. 3B). Importantly, MLV-GFP replicated in cultured NIH 3T3 cells with kinetics that were nearly the same as those of wild-type MoMLV (Fig. 3C). We measured MLV-GFP spreading through a monolayer by monitoring the growth of foci of MLV-GFP-infected cells, commencing observation when individual MLV-GFP-infected cells were detected in monolayers ∼12 h after infection. As was the case with VSV infection, MoMLV infection of cells that did or did not express the human or mouse tetherin protein yielded approximately equal numbers of GFP-positive cells, suggesting that MoMLV entry and gene expression were not affected by tetherin. Because the MoMLV replication rate is lower than that of VSV, we recorded MLV-GFP infections over the course of 72 h following the initial detection of infected cells. In contrast to the pattern observed with VSV-GFP, MLV-GFP appeared to spread only to adjacent cells, even under unrestricted growth conditions (Fig. 3D, left column; Movies S3 and S4, upper left panels).

**FIG 4:**
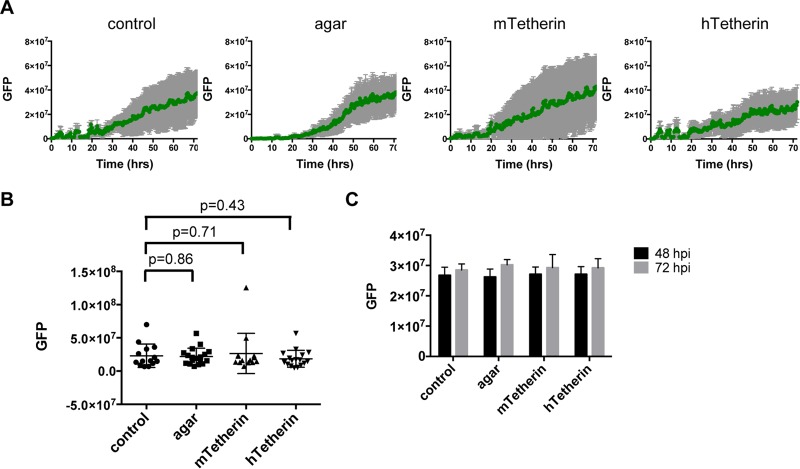
Quantitation of MoMLV spread in a cell monolayer. (A) Quantitation of MLV spread in region 1, as described in the legend to Fig. 2A. (B) MLV-GFP integrated intensity in region 1 at 48 h postinfection. Each dot represents one movie, with each horizontal line showing the average for each population and error bars indicating 1 standard deviation. *P* values were determined by Student's *t* test. (C) Quantitation of total GFP intensity in each field (∼2.5 mm × 2 mm) as described in the legend to Fig. 2C.

Notably, the growth rates of MLV-GFP-infected foci appeared to be similar in the presence and absence of an agar overlay (Fig. 3D; Movies S3 and S4, upper right panels), suggesting that cell-free dissemination of virions does not contribute substantially to MoMLV spread through a monolayer. Moreover, expression of either mouse or human tetherin did not noticeably affect the growth of an MLV-GFP focus (Fig. 3D; Movies S3 and S4, lower panels), indicating that tetherin does not inhibit MLV spread between cells that are in direct contact. Quantitation of MLV-GFP infection by the aforementioned methods confirmed that the growth of foci of MLV-GFP infection was not measurably inhibited by either an agar overlay or tetherin expression (Fig. 4A and B), and unlike the results with VSV-GFP, this was also true for the total GFP intensity in a larger microscopic field (Fig. 4C). Thus, while MoMLV generated significant titers of diffusible cell-free virions in monolayer cultures, the apparently greater efficiency with which the virus spread from infected cells to proximal rather than distal uninfected cells means that direct cell-to-cell transmission constitutes the dominant mode of MoMLV propagation in cell monolayers *in vitro*.

### Generation of mice with ubiquitous or tissue-specific tetherin expression.

**FIG 5:**
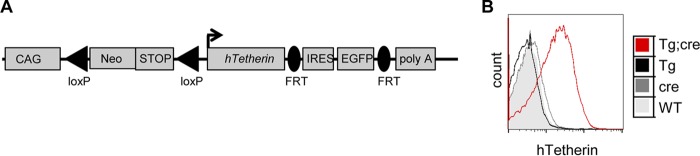
Generation of inducible hTetherin-transgenic mice. (A) Schematic of the targeting cassette. A human tetherin transgene (Tg) was inserted into the pCTV targeting vector 3′ to the loxP (triangles)-flanked transcription stop signal (STOP) and 5′ to an FRT-flanked internal ribosome entry site (IRES)-EGFP sequence. The entire cassette was targeted to the mouse *Rosa26* locus. (B) Peripheral blood leukocytes were stained for human tetherin expression and analyzed by flow cytometry. Mice carried both the human tetherin and cre transgenes (red) or either transgene alone (black and gray lines). Wild-type animals were also analyzed (gray filled histogram).

Although cultured cell monolayers have been considered models of the simplest tissues, neither they nor any other cocultivation scenario can recapitulate the anatomical and fluid-dynamic complexity of the environment confronting a virus as it replicates in an animal host. Therefore, we next sought to determine the effects of tetherin and cell-free virions on MoMLV dissemination in an animal. To determine the effects of tetherin expression *in vivo* without the confounding effects of the expression of many hundreds of ISGs, we generated a mouse line in which the expression of an hTetherin transgene could be induced in a cell-type-dependent manner in the absence of IFN. Specifically, a lox-STOP-lox cassette was positioned between a constitutive promoter (CAG) and an hTetherin cDNA sequence (Fig. 5A), preventing hTetherin expression unless the transgene encountered Cre recombinase. Mice that were hemizygous for the inducible hTetherin transgene were crossed with mice hemizygous for a cytomegalovirus (CMV) promoter-driven Cre recombinase transgene (CMV-cre), generating offspring that were either wild type (WT), carried the hTetherin transgene alone (Tg), carried the CMV-cre transgene alone (cre), or carried both transgenes (Tg; cre). Importantly, hTetherin expression was detected only in animals carrying both the hTetherin and CMV-cre transgenes (Fig. 5B).

**FIG 6:**
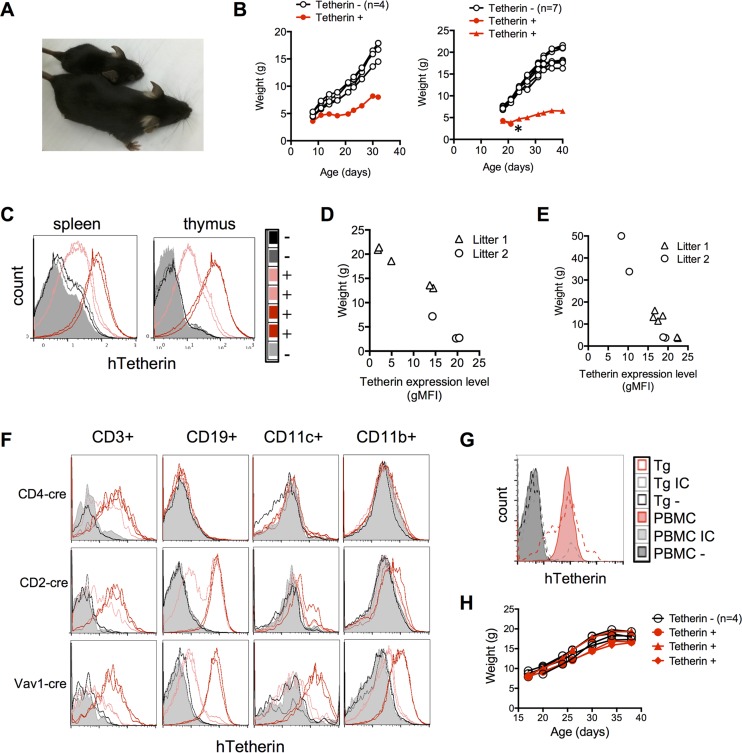
Characterization of mice carrying an inducible hTetherin transgene. (A) Typical appearance of mouse littermates at 21 days old. The smaller animal carried both the hTetherin and CMV-cre transgenes, while the larger animal carried only the hTetherin transgene. (B) Weights of littermates that did or did not constitutively and ubiquitously express hTetherin over time. The two charts represent two different litters. The asterisk indicates an animal that died. (C) FACS analysis of hTetherin expression on splenocytes and thymocytes from one litter of mice. A “+” indicates the presence of both the hTetherin and CMV-cre transgenes. (D and E) Weights and mean fluorescence intensities (gMFI) of hTetherin expression on splenocytes from mice generated by hTetherin × CMV-cre (D) and hTetherin × Zp3-cre (E) crosses for 2 litters of mice at ∼7 weeks of age. (F) hTetherin expression (colored lines) on splenocytes from hTetherin Tg mice crossed with mice bearing the CD4-cre, CD2-cre, or Vav1-cre transgene, after gating for T cells (CD3), B cells (CD19), dendritic cells (CD11c), and macrophages (CD11b). Data for control mice (hTetherin Tg alone, without cre) are depicted by gray and black histograms. (G) Human PBMCs stimulated with IFN-α were either left unstained (black filled histogram), stained with an isotype control (IC) antibody (gray filled histogram), or stained for hTetherin (red filled histogram). Splenic lymphocytes from mice carrying both the hTetherin and Vav1-cre transgenes were left unstained (black dashed line), stained with an isotype control antibody (gray dashed line), or stained for hTetherin (red dashed line). (H) Weights of mice bearing either the uninduced hTetherin Tg alone or a Vav1-cre-induced hTetherin Tg over time.

Mice homozygous for the hTetherin transgene were mated to mice that were hemizygous for CMV-cre, resulting in litters in which ∼50% of the offspring carried both hTetherin and CMV-cre, while the remainder carried hTetherin in its uninduced configuration. Most of these litters contained one or more mice that were noticeably smaller than their littermates, some of which died prematurely (Fig. 6A and B). Flow cytometric analysis of splenocytes and thymocytes from mice that carried both the hTetherin and CMV-cre transgenes revealed various levels of hTetherin expression (Fig. 6C). In fact, there was a striking inverse correlation between the level of tetherin expression and body weight (Fig. 6D). A similar analysis of the progeny of matings between hTetherin and zona pellucida 3 (*Zp3*) promoter-driven cre (Zp3-cre) transgenic mice revealed ubiquitous hTetherin transgene expression in the offspring of females carrying both transgenes, as expected. Notably, the relationship between hTetherin expression and body weight was the same as that observed for the CMV-cre-expressing mice, indicating that the effect was specific to tetherin and did not require ongoing cre expression (Fig. 6E). Examination of postmortem mouse tissues revealed no specific pathology in hematopoietic or other tissues that could explain the runt development that was associated with ubiquitous hTetherin expression.

By crossing mice carrying the inducible hTetherin transgene and mice carrying cre driven by the CD4, CD2, or Vav1 promoter (CD4-cre, CD2-cre, or Vav1-cre), we generated mice specifically expressing hTetherin only in T cells (CD4-cre), in both T and B cells (CD2-cre), or in all hematopoietic cells (Vav1-cre), respectively (Fig. 6F). The level of hTetherin Tg expression on mouse cells was then compared to the level of endogenous tetherin induced by IFN-α treatment of human peripheral blood mononuclear cells (PBMCs) (Fig. 6G). Although the level of hTetherin expression on mouse cells was more variable, the majority of the cells expressed hTetherin at a level nearly identical to that found on the IFN-α-stimulated human PBMCs (Fig. 6G). Importantly, expression of hTetherin on T and B cells, or indeed on all hematopoietic cells, did not have the same adverse effects on growth that were observed with ubiquitously expressed hTetherin (Fig. 6H). Together, these data show that ubiquitous expression of hTetherin profoundly reduces the fitness of mice, with marked dose-dependent adverse effects on growth and survival, perhaps explaining why tetherin has evolved to be IFN inducible in most cell types. However, hTetherin expression on hematopoietic cells was not responsible for these defects.

### Tetherin expression in hematopoietic cells reduces MoMLV plasma viremia.

**FIG 7:**
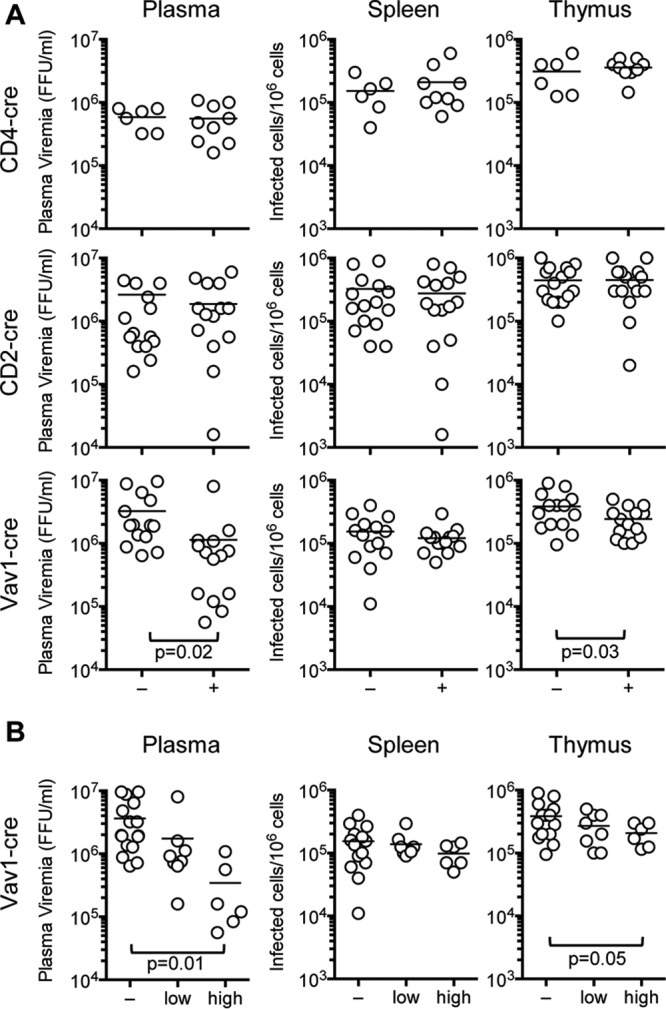
Effect of hematopoietic cell hTetherin expression on MoMLV replication *in vivo*. (A) MoMLV loads in the plasma (FFU per milliliter) and the spleen and thymus (numbers of infected cells per 10^6^ cells) 14 days after infection of mouse neonatal littermates bearing the hTetherin Tg and the indicated cre inducers (+) or the hTetherin Tg alone (−). (B) MoMLV loads in mice bearing the hTetherin Tg alone (−) or the hTetherin Tg and Vav1-cre, grouped according to hTetherin expression level (low or high).

Analysis of viral infection in mice in which hTetherin expression was induced in all tissues would be complicated by the confounding growth and survival defects. Therefore, we limited our viral infection studies to mouse lines in which hTetherin expression was induced only in subsets of hematopoietic cells. Specifically, neonatal mice harboring the hTetherin transgene and expressing cre in (i) T cells only, (ii) T and B cells, or (iii) all hematopoietic cells were infected with MoMLV. The numbers of infected splenocytes and thymocytes, as well as plasma viremia, were measured 2 weeks after infection. For mice expressing hTetherin either in T cells alone or in both T and B cells, the numbers of infected cells and levels of plasma viremia were indistinguishable from those of control (cre-negative) littermates that lacked hTetherin expression (Fig. 7A). However, expression of hTetherin in all hematopoietic cells caused a significant reduction (*P* = 0.02) in plasma viremia (Fig. 7A). Notably, the numbers of infected splenocytes were indistinguishable in hTetherin-expressing and control animals, while the numbers of infected thymocytes were only marginally reduced as a consequence of hTetherin expression (Fig. 7A).

Because the hTetherin levels varied among mice in which its expression was induced (Fig. 6C and F), we segregated hTetherin-expressing animals into those with high and low expression levels. This analysis revealed that the hTetherin expression level inversely correlated with plasma viremia (Fig. 7B). There was a marginal, statistically ambiguous inverse correlation between the tetherin expression level and the infected thymocyte number, and there was no apparent effect of tetherin on infection of splenocytes (Fig. 7B).

**FIG 8:**
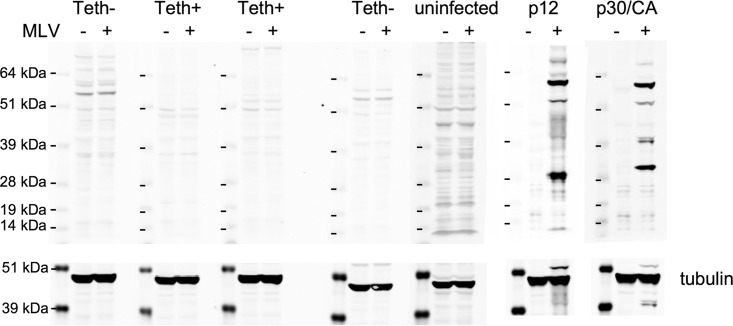
Lack of anti-MoMLV antibodies during acute infection. Plasmas from hBst2 × Vav1-cre littermates infected with MoMLV were isolated at 14 days postinfection and used to probe lysates of uninfected (−) or MoMLV-infected (+) NIH 3T3 cells. All animals were either hTetherin transgene hemizygous alone (Teth−) or hTetherin transgene hemizygous and Vav1-cre hemizygous (Teth+). The same lysates were also probed with plasma from an uninfected animal as well as with antibodies to either the MLV p12 or p30/CA protein. Membranes were probed with an antibody against tubulin as a loading control.

In addition to exerting a direct antiviral effect by inhibiting virion release, hTetherin has been shown to facilitate antibody-dependent cell-mediated cytotoxicity (ADCC) of infected cells *in vitro* by causing antigen retention on the surfaces of those infected cells (24). In principle, therefore, reduced plasma viremia may potentially be the result of a shorter infected cell life-span due to ADCC. We therefore determined whether anti-MoMLV antibodies were detectable in mice during acute infection at the time at which the plasma viremia levels and infected cell numbers were determined. A mouse litter derived from mating hTetherin transgenic and Vav1-cre mice was infected with MoMLV and monitored for the presence of anti-MoMLV antibodies. None of the mice had detectable levels of anti-MoMLV antibodies in the blood at 2 weeks postinfection (Fig. 8), excluding antibody-mediated effects as an explanation for the influence of tetherin on plasma viremia at this time point.

**FIG 9:**
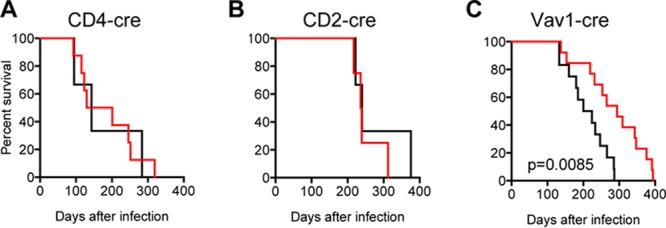
Effect of hematopoietic cell hTetherin expression on MoMLV pathogenesis. Mice born from matings between mice homozygous for the hTetherin transgene and mice expressing either CD4-cre (A), CD2-cre (B), or Vav1-cre (C) were infected with MoMLV and monitored for morbidity and mortality. Black, no tetherin expression; red, cre-induced tetherin expression.

To determine the effects of tetherin on MoMLV pathogenesis, parallel cohorts of mice bearing the inducible hTetherin transgene in its uninduced state versus those also carrying either a CD4-cre, CD2-cre, or Vav1-cre transgene were maintained for approximately 1 year, and leukemia-induced deaths were monitored. Mice expressing hTetherin in T cells only (CD4-cre) or in T and B cells (CD2-cre) exhibited no difference in MoMLV-induced mortality compared to that of littermates carrying the uninduced hTetherin transgene (Fig. 9A and B). In contrast, animals expressing hTetherin on all hematopoietic cells exhibited a significant (*P* = 0.0085) delay in MoMLV-induced mortality (Fig. 9C). Thus, the inhibitory effect of hTetherin on the generation of cell-free virus correlated better with its ability to delay leukemia than did its lack of effect on the number of MoMLV-infected splenocytes or thymocytes.

## DISCUSSION

Although neither VSV (4) nor MoMLV is inhibited by tetherin at early steps in the replication cycle, the ability of tetherin to inhibit the release of virions from infected cells into the extracellular milieu of cultured cells is well established. However, whether tetherin can inhibit the dissemination of infection among cells in contact with each other, as would occur in solid tissues, is less well understood. We developed a live-cell imaging assay by which we could monitor multicycle VSV and MoMLV replication in the simplest two-dimensional model of a tissue, namely, a cell monolayer. The results of these experiments clearly showed that neither VSV nor MoMLV spreading between adjacent cells was greatly inhibited by tetherin expression.

The inability of agar or tetherin to inhibit the spread of MoMLV within a monolayer and the lack of an effect of tetherin on the numbers of MoMLV-infected cells in spleen and thymus tissues suggest that the majority of virus spread in monolayer cultures and in tissues *in vivo* occurs between neighboring cells. This finding is consistent with previous studies indicating that transfer of MLV from transfected cells to recipient cells in a single cycle was not inhibited by methyl cellulose (25). Nevertheless, expression of hTetherin in hematopoietic cells caused a significant reduction in MoMLV plasma viremia and, crucially, a delay in the onset of disease and mortality. These findings suggest a key role for cell-free virions in the dissemination of viral infection *in vivo*.

In mice, tetherin is expressed on a small subset of cells in the absence of IFN or another inductive signal (19). Presumably, tetherin would present a more effective antiviral defense if it were ubiquitously and constitutively expressed. However, our data suggest that a dramatic fitness cost is associated with such expression, though it is not yet clear why. When constitutive hTetherin expression was limited to hematopoietic cells, or subsets thereof, mice developed normally, suggesting that adverse effects of hTetherin on growth and survival are due to its expression on nonhematopoietic cell types.

Tetherin expression on all hematopoietic cells reduced plasma viremia and delayed disease, while expression on lymphocytes alone did not. This finding strongly suggests that hematopoietic cells other than lymphocytes constitute a major source of plasma virions, even though MoMLV induces tumors primarily in lymphocytes (26, 27). Importantly, however, the types of tumors induced by MoMLV appear to be determined not by the inherent susceptibility of a particular hematopoietic lineage to MoMLV infection but by the U3 sequences. Indeed, tumors of myeloid lineage cells (erythroleukemias and monocytoid leukemias) are induced if U3 sequences are exchanged with those from MLV strains that induce this type of leukemia. Moreover, promonocyte tumors occur at a low frequency in mice infected with unmodified MoMLV (28–31). Thus, it is unsurprising that nonlymphocyte hematopoietic cells appear to represent a significant source of plasma virions.

Despite the above considerations, it is formally possible that hTetherin expression on nonlymphoid hematopoietic cells leads indirectly to a reduction in viral yield from other cells (e.g., lymphocytes). The consequences of tetherin-induced virion retention on infected cell surfaces may be more complex than simply restricting infection to proximal cells and directly reducing the cell-free virion titer. Accumulation of entrapped virions can also have indirect antiviral effects, including tetherin-driven stimulation of proinflammatory signaling pathways (32–35), enhancement of antiviral immune responses (36, 37), and enhancement of ADCC (24, 38). Given that we did not detect anti-MoMLV antibodies concomitant with reductions in plasma viremia and that equal numbers of infected cells were measured in hTetherin-expressing and non-hTetherin-expressing mice, it is unlikely that ADCC contributed to the effects that we observed. In a Friend MLV (FV) infection model, the tetherin allele found in B6 mice was observed to promote cellular antiviral immune responses, including increased NK and T cell activity, when the allele was expressed in combination with the MHC H-2^b^ haplotype found in B6 mice (36, 37). At early time points of infection (5 to 7 days postinfection), there was no apparent tetherin-dependent effect on either plasma viremia or the number of infected splenocytes, but increased dendritic cell (DC) and NK cell activation was observed in WT compared to tetherin-deficient mice (37). Conversely, at 2 weeks postinfection, decreases in plasma viremia and infected splenocytes were observed in WT compared to tetherin-deficient mice (36), as would be expected in the presence of potent antiviral cellular immune responses. In contrast, we observed a decrease in plasma viremia, but not in infected splenocytes or thymocytes, in tetherin-transgenic mice, suggesting that a different mechanism is operative. Among several differences in the two studies, a key difference is that MoMLV is a single replication-competent virus, while FV is an immunosuppressive complex of replication-defective and -competent viruses that may easily lead to the elicitation of different host responses to infection. The reduction in plasma viremia observed herein correlated with tetherin surface expression levels, which is more consistent with a direct tethering mechanism in infected cells as the means by which tetherin prevents virus dissemination and pathogenesis.

Although retroviruses appear to spread *in vitro* primarily via direct cell-to-cell transmission in a manner that is not affected by tetherin, many enveloped viruses, including some retroviruses, such as HIV-1, have evolved tetherin antagonists. This fact, in addition to observations reported herein, indicate an important role for tetherin in the host defense against retroviruses. Moreover, our current findings suggest that both cell-to-cell transmission and cell-free virion particles are key mediators of viral dissemination *in vivo*.

## MATERIALS AND METHODS

### Generation of transgenic mice carrying an inducible hTetherin transgene.

The human tetherin gene was cloned into the AscI site of pCTV (Addgene plasmid 15912) and targeted to the *Rosa26* locus (Fig. 5) of C57BL/6J mice to generate the mouse line B6.*Gt*(*ROSA*)^*26Sortm1*(*CAG-BST2*)*Bsz*^/J. Mice that were hemizygous for the transgene *Gt*(*ROSA*)^*26Sortm1*(*CAG-BST2*)*Bsz*^ (abbreviated *hBST2*) were intercrossed to generate mice homozygous for *hBST2* and then mated to mice hemizygous for a Cre recombinase transgene under the control of one of several promoters (all from The Jackson Laboratory), as follows: CMV-cre, Zp3-cre, CD4-cre, CD2-cre, or Vav1-cre. Mice were genotyped by PCR for the *hBST2* transgene or the wild-type allele by use of the following primers (5′ to 3′): RL267, GGCATCTACTTCGTATGACTATTGCAGA (*hBST2* forward); RL268, GAGGCCCAGCAGCACAATCAGCAGCT (*hBST2* reverse); RL286, AGTTCTCTGCTGCCTCCTGGCTTCT (WT forward); and RL340, CTGAAAATTAAGGATCAAGGCAAAGGAT (WT reverse). The *hBST2* PCR yields a 522-bp product, and the WT reaction yields a 720-bp product. The CMV-cre, Zp3-cre, and CD4-cre transgenes were amplified by use of JAX primers oIMR1084 (GCGGTCTGGCAGTAAAAACTATC) and oIMR1085 (GTGAAACAGCATTGCTGTCACTT), yielding a 100-bp product. The CD2-cre and Vav1-cre transgenes were amplified by use of JAX primers oIMR9266 (AGATGCCAGGACATCAGGAACCTG) and oIMR9267 (ATCAGCCACACCAGACACAGAGATC), yielding a 236-bp product. Mice were housed in a pathogen-free facility and maintained in accordance with Institutional Animal Care and Use Committee (IACUC) guidelines. All animal studies were approved by the Rockefeller University IACUC (protocol number 15785-H) in accordance with the Animal Welfare Act (USDA) and the Public Health Services Policy of the Office of Laboratory Animal Welfare (OLAW).

### Tetherin-expressing cell lines.

NIH 3T3 (ATCC; CRL-1658) cells were stably transduced with the empty pLHCX (Clontech) vector or with pLHCX containing either mouse or human tetherin cDNA. The cells expressing mouse tetherin have been described previously (21). For the human tetherin-expressing cell lines, single-cell clones were selected with hygromycin and analyzed by flow cytometry for hTetherin expression (phycoerythrin [PE]-anti-CD317/Bst2; BioLegend).

### Viruses and infections.

The MoMLV-GFP construct was generated by inserting an FMDV 2A sequence followed by the enhanced GFP (EGFP) sequence between the 3′ end of *env* and the long terminal repeat (LTR) in the infectious plasmid clone of MoMLV, pNCS (39). Specifically, a PmeI site and a NotI site were introduced into pNCS, a MoMLV molecular clone, between the 3′ end of *env* and the 3′ LTR. Subsequently, the Env stop codon was removed and an FMDV 2A sequence flanked by PmeI and EcoRI sites was inserted. Finally, the EGFP gene was inserted between the EcoRI and NotI sites, resulting in pNCS ENV-2A-GFP. MoMLV and MoMLV-GFP stocks were generated by transfecting NIH 3T3 cells with proviral plasmids. Harvested supernatant was filtered, and viral stocks were titered on NIH 3T3 cells by a focal immunoassay as described previously (21). For *in vivo* MoMLV infections, measurements of plasma viremia and infected cell numbers in spleen and thymus tissues were performed as previously described (21). VSV carrying an EGFP gene (40) was kindly provided by Kartik Chandran (Albert Einstein College of Medicine, New York, NY). VSV-GFP stocks were generated in Vero cells (ATCC; CCL-81) and titrated by plaque assay.

### Western blot analysis.

Uninfected or MoMLV-infected NIH 3T3 cells were lysed in RIPA buffer (150 mM NaCl, 50 mM Tris, pH 7.4, 0.1% SDS, 1 mM EDTA, 1% Igepal, 1% sodium deoxycholate). Plasma samples were obtained from blood collected from animals at 14 days post-MoMLV infection and diluted 1:100 in phosphate-buffered saline (PBS). Plasma antibody was detected with an anti-mouse IgG secondary antibody (Li-Cor). The anti-p12 (CRL-1890) and anti-p30 (CRL-1912) antibodies were obtained from ATCC.

### Live-cell imaging and quantitative analysis.

All live-cell imaging was performed with a VivaView FL incubator fluorescence microscope imaging system (Olympus). Cells were infected with VSV-GFP or MoMLV-GFP at an MOI of 0.0001 or 0.001, respectively, and transferred to the VivaView incubator. Single infected, GFP-positive cells were centered in a field, and images were acquired at 5- to 15-min intervals for 24 to 72 h. Images were analyzed with MetaMorph software (Molecular Devices). Images taken at a magnification of ×4 were acquired on an Evos FL microscope and quantitated with ImageJ software.

### Flow cytometry.

For fluorescence-activated cell sorter (FACS) analysis of mouse primary cells, single-cell suspensions were made from spleen, thymus, or peripheral blood, resuspended in red blood cell lysis buffer (150 mM NH_4_Cl, 10 mM KHCO_3_, and 0.05 mM EDTA), washed in FACS buffer (PBS, 0.2% bovine serum albumin [BSA]), incubated with purified anti-CD16/CD32 (Fc Block), and then stained with the following antibodies (all from BD Biosciences): peridinin chlorophyll protein (PerCP)–Cy5.5–anti-CD3, V450–anti-CD19, allophycocyanin (APC)–Cy7–anti-CD11b, APC–anti-CD11c, PerCP–Cy5.5–anti-CD4, and V450–anti-CD8. Adherent cell lines were harvested in PBS plus 5 mM EDTA, pelleted, and stained in FACS buffer. Human PBMCs were stimulated with 1,000 U/ml human IFN-α for 24 h. Human tetherin expression on primary cells and stable cell lines was detected with PE–anti-human CD317/Bst2 (BioLegend), and PE-mouse IgG1 (BioLegend) was used as an isotype control. Dead cells were excluded by DAPI (4′,6-diamidino-2-phenylindole) staining. All data were acquired on an LSR II flow cytometer (Becton Dickinson) and analyzed with FlowJo software (Tree Star).

## Supplementary Material

Supplemental material
