# Supplementary material for: Tetherin Inhibits Cell-Free Virus Dissemination and Retards Murine Leukemia Virus Pathogenesis
Source: J Virol. 2017 May 26;91(12):e02286-16. doi: 10.1128/JVI.02286-16 (PMC5446635; doi:10.1128/JVI.02286-16)
Supplement: Supplemental material [file supp_91_12_e02286-16__index.html]

Supplemental material 

# Tetherin Inhibits Cell-Free Virus Dissemination and Retards Murine Leukemia Virus Pathogenesis

## Supplemental material

- Supplemental file 1 -

  Movie S1 (Examples of live imaging of VSV-GFP spreading in a monolayer.)

  MOV, 1.2M
- Supplemental file 2 -

  Movie S2 (Examples of live imaging of VSV-GFP spreading in a monolayer [part 2].)

  MOV, 1.2M
- Supplemental file 3 -

  Movie S3 (Examples of live imaging of MLV-GFP spreading in a monolayer.)

  MOV, 1.3M
- Supplemental file 4 -

  Movie S4 (Examples of live imaging of MLV-GFP spreading in a monolayer [part 2].)

  MOV, 1.3M
- Supplemental file 5 -

  Supplemental Movie Legends.

  PDF, 36K
