# Supplementary material for: Tetherin Inhibits Cell-Free Virus Dissemination and Retards Murine Leukemia Virus Pathogenesis
Source: J Virol. 2017 May 26;91(12):e02286-16. doi: 10.1128/JVI.02286-16 (PMC5446635; doi:10.1128/JVI.02286-16)
Supplement: Supplemental material [file JVI.02286-16_zjv999182650s5.pdf]

Supplemental Movie Legends

Movies S1 and S2. Examples of live imaging of VSV-GFP spreading in a monolayer.
NIH 3T3 cells were infected with VSV-GFP at MOI 0.0001 as in Fig. 1A and individual infected (GFP-positive) cells were centered in a field of observation and images were recorded every 10 minutes for 2 days.
(upper left) NIH 3T3 cells were infected and imaged
(upper right) NIH 3T3 cells were infected and imaged with an agar overlay applied to the cells following infection.
(lower left) NIH 3T3 cells stably expressing mTetherin were infected and imaged.
(lower right) NIH 3T3 cells stably expressing hTetherin were infected and imaged.

Movies S3 and S4. Examples of live imaging of MLV-GFP spreading in a monolayer.
NIH 3T3 were infected with MLV-GFP at MOI 0.01 as in Fig. 3A and individual infected (GFP-positive) cells were centered in a field and images were recorded every 15 minutes for 3 days.
(upper left) NIH 3T3 cells were infected and imaged
(upper right) NIH 3T3 cells were infected and imaged with an agar overlay applied to the cells following infection.
(lower left) NIH 3T3 cells stably expressing mTetherin were infected and imaged.
(lower right) NIH 3T3 cells stably expressing hTetherin were infected and imaged.
